# Case Report: ASCENIV use in three young children with immune abnormalities and acute respiratory failure secondary to RSV infection

**DOI:** 10.3389/fimmu.2023.1154448

**Published:** 2023-09-14

**Authors:** Constance Bindernagel, Shannon Sotoudeh, Minh Nguyen, Gene Wetzstein, Panida Sriaroon, Jolan Walter

**Affiliations:** ^1^ Department of Pediatrics, Division of Allergy and Immunology, University of South Florida Morsani College of Medicine, Tampa, FL, United States; ^2^ Department of Allergy and Immunology, Johns Hopkins All Children’s Hospital, Saint Petersburg, FL, United States; ^3^ ADMA Biologics (United States), Ramsey, NJ, United States

**Keywords:** RSV, ASCENIV, pediatric, immunodeficiency, immune dysregulation, respiratory viral infections, acute respiratory failure, IVIG

## Abstract

Respiratory syncytial virus (RSV) is the most common etiology of bronchiolitis in young children. While most children clinically improve with care at home, RSV is the leading cause of hospitalization among infants aged 12 months or less. Common modalities of treatment for children with immune dysregulation include respiratory support and best supportive care, which may include immunoglobulin therapy. All immunoglobulin therapies adhere to Food and Drug Administration (FDA) - established standards for antibodies against measles, polio, and diphtheria, but there are no required standards for problematic respiratory viral pathogens, including RSV and others. ASCENIV is an approved IVIG that is manufactured from blending normal source plasma with plasma from donors that possess high antibody titers against RSV and other respiratory pathogens of concern. ASCENIV was developed, in part, to the unmet need that exists in immunocompromised patients who lack sufficient antibodies against problematic viral pathogens. ASCENIV is not a currently approved treatment for severe RSV and other viral infections. There is a lack of research regarding its potential benefits in the acute treatment period for RSV and in the pediatric population. Therefore, this case series was developed to describe real-world experiences of ASCENIV use in this less well studied clinical scenario. This case series reviews three pediatric patients ≤ 5 years of age with immune dysregulation and who were severely ill with RSV. Despite receiving best supportive care, and standard immunoglobulin therapy for some, the patients’ clinical status continued to decline. All patients received ASCENIV in an intensive care setting. Each patient had ultimately recovered due to the various medical interventions done. This case series demonstrated that ASCENIV (500mg/kg) administration may have contributed to the treatment outcomes of a less well studied age-cohort of patients. In addition, no adverse side effects were observed after ASCENIV administration. Further analysis of the benefits of ASCENIV for the acute and preventative treatment in patients younger than 12 years of age with immune dysregulation should continue to be explored.

## Introduction

RSV infects nearly all children by 2 years-of-age (1). Infectivity in the United States has historically peaked between mid-October to early May (2), however, social restrictions in response to the COVID-19 pandemic interrupted the normal respiratory virus circulation. In 2021 and 2022, RSV infections peaked in the summer months (3), indicating highly unpredictable seasonality.

RSV is the most common etiology of bronchiolitis in young children. Methods of testing for RSV include nasopharyngeal aspirate, real time polymerase chain reaction (RT-PCR), direct fluorescence antibody test, viral culture, and rapid antigen test (4). While most children clinically improve with care at home, RSV is the leading cause of hospitalization among infants aged 12 months and less (5). Common modalities of treatment for children with immune dysregulation include respiratory support and best supportive care, which may include immunoglobulin therapy. All immunoglobulin therapies adhere to FDA - established standards for antibodies against measles, polio, and diphtheria, but there are no required standards for problematic respiratory viral pathogens, including RSV and others.

## Methods

Chart reviews of three children with severe courses of RSV infections were performed. All patients required mechanical ventilator or high frequency oscillatory ventilation (HFOV) support. The first two patients underwent a primary immune evaluation as there was concern for a new presentation of immune dysregulation. The evaluation included extended immune phenotyping for lymphocyte subsets (T, B, NK and naïve/memory T cells), immunoglobulin panel, pneumococcus titers, diphtheria and pertussis titers, lymphocyte proliferation to mitogens and antigens, and sequencing for 407 primary immunodeficiency genes (Invitae Primary Immunodeficiency Panel). The third patient had recently been diagnosed with specific antibody deficiency and was in the process of beginning immunoglobulin replacement. All three patients had a form of immune dysregulation and received one dose of ASCENIV at 500 mg/kg in the acute care setting.

## Case description

**Figure 1:**
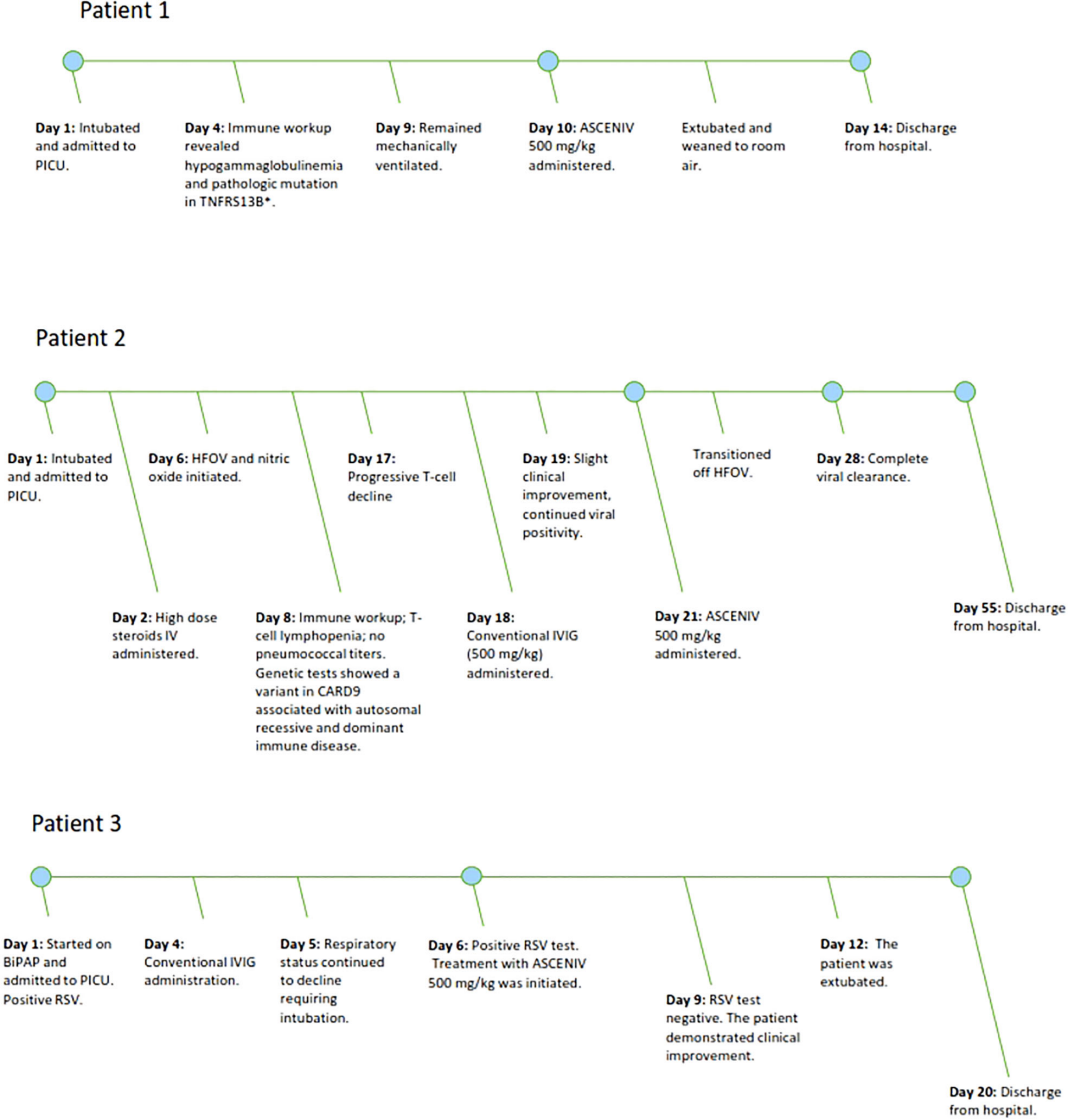
**Figure 1** demonstrates the clinical timeline of three pediatric patients with severe courses of RSV lower respiratory tract infections. The timeline demonstrates the point in which ASCENIV 500 mg/kg was given during each patient’s hospitalization.

Patient demographics, past medical history, primary immune evaluation, genetic testing (if applicable), relevant laboratory values, and clinical courses are provided in **Table 1**. **Figure 1** presents a graphical depiction of patients’ clinical course and timeline.

**Table 1:** Clinical course of three pediatric patients who received ASCENIV.

	Patient 1	Patient 2	Patient 3
**Demographics**	Male, 12-months-old	Male, 15-month-old	Female, 5-years-old
**Past medical/respiratory history**	Eczema, craniosynostosis Several hospital admissions for severe viral RTIs, including a previous RSV infection.	Bilateral ear infections Frequent viral upper RTIs Diagnosed with RSV lower RTI four months prior to hospitalization	Cerebellar ataxia (genetic form), global developmental delay, SAD Multiple upper RTIs, two PICU admissions for viral pneumonia Moderate persistent asthma
**Primary immune evaluation/Relevant laboratory values**	The patient is fully immunized for age Low immunoglobulin (Ig): IgG and IgG selective subclass deficiency consistent with hypogammaglobulinemia Out of range results: Hospital/PICU Day 4 IgG=190 mg/dL IgG1 = 129 mg/dL IgG3 = 14 mg/dL There were not out of range results for total lymphocytes.	The patient is fully immunized for age On initial evaluation, 0/23 pneumococcal titers and progressive T-cell lymphopenia Out of range results, absolute lymphocytes (cells/µL): Hospital/PICU Day 8 * Total lymphocytes: 1073 cells/µL Hospital/PICU Day 17 * Total lymphocytes: 773 cells/µL Hospital/PICU Day 28 * Total lymphocytes: 3495 cells/µL^a^ RSV: positive on Days 0 and 19; negative on Days 8, 28, and 34 Rhino/enterovirus: positive on Days 0, 8, and 19; negative on Day 34.	The patient is fully immunized for age On initial immune evaluation, 0/23 pneumococcal serotypes. After vaccination, had 14/23 serotypes. 20 months later, repeat testing demonstrated 5/23 serotypes. After repeat vaccination, had 1/23 serotypes.
**Genetic testing**	On pathologic heterozygous mutation TNFRSF13B (TACI) in p.Cys104Arg Additional variants of unknown significance included ARMC4, CD79B, HPS3, JAK3.	One pathological heterozygous mutation in CARD9 in p.Val13Ile Additional variants of unknown significance included C7, EFL1, IL12RB2, SMAHD1.	In 6/2018, genomic testing showed chromosomal abnormality (18p11.22 and 13 mutation) 11/2018, heterozygous for the *De Novo* p.Q306R VUS in the POU4F1 gene.
**Clinical course**	* Tachypnea, increased work of breathing, positive for RSV * Acute respiratory decompensation, intubation * Admitted to the PICU * Broad-spectrum antibiotics for suspected superimposed bacterial pneumonia	* Hypoxia, increased work of breathing and grunting, positive for RSV and rhinovirus/enterovirus * Acute respiratory decompensation, intubation * Admitted to the PICU * Persistent viral positivity * Diagnosed with superimposed bacterial and ventilator associated pneumonia secondary to Moraxella catarrhalis and MRSA * High-dose IV steroids * HFOV, nitric oxide * Broad-spectrum antibiotics with PJP prophylaxis * 500 mg/kg conventional IVIG	* Respiratory distress, positive for RSV * Required BiPAP * Admitted to the PICU * Persistent respiratory failure requiring intubation * RSV positivity persisted * 600 mg/kg of conventional IVIG
**ASCENIV initiation (day of hospital course)**	ASCENIV 500 mg/kg Day 10	ASCENIV 500 mg/kg Day 21	ASCENIV 500 mg/kg Day 6
**Clinical course after ASCENIV**	Respiratory status improved; was extubated, weaned to room air Discharged on Day 14 Has remained on monthly Ig therapy for hypogammaglobulinemia	Respiratory status improved; transitioned off HFOV support to conventional ventilation Steroids tapered off; improved T-cell populations. Consecutive PCR clearance of RSV and rhino/enterovirus PJP prophylaxis discontinued as lymphocyte count normalized Discharged on Day 55 Was to remain on monthly Ig therapy due to concern for SAD	Respiratory status improved; was extubated RSV Rapid detection became negative. Discharged on Day 20 Has remained on monthly Ig therapy

MRSA, methicillin resistant staphylococcus aureus; VUS, variant(s) of unknown significance; SAD, specific antibody deficiency, Ig therapy, immunoglobulin replacement therapy

Reference ranges: 10-12 months: IgG 594 mg/dL (294-1069), 13-23 months: IgG 679 mg/dL (345-1213), 4-5 years old: IgG mg/dL 780 (463-1236) based off Harriet Lane Values, 22 edition, page 379. Total lymphocytes: 2.8-12.3 cells/µL.

Patient 1 was a 12-month-old boy with a pertinent history of three previous hospitalizations for severe respiratory infections, including RSV (**Table 1**). On the most recent hospital admission, the patient presented with tachypnea and increased work of breathing. He tested positive for RSV on viral RT-PCR. Patient experienced acute respiratory decompensation, was intubated, and admitted to the pediatric intensive care unit (PICU) for acute respiratory failure. The patient received broad-spectrum antibiotics for suspected superimposed bacterial pneumonia but continued to remain ventilator dependent.

Primary immune evaluation (Day 4) results were consistent with hypogammaglobulinemia, with a total IgG of 190 mg/dL. Genetic testing revealed one pathologic heterozygous mutation of p.Cys104Arg in the TNFRSF13B (TACI) gene. This specific mutation has been reported to be associated with autosomal recessive common variable immunodeficiency (CVID) (6). One heterozygous mutation was found to cause an increased risk of CVID and to possibly induce a CVID-like picture in mice models (7). Mutations at p.Cys104Arg have been described as one of the most common genetic mutations found in CVID (8).

Considering the patient’s immune abnormality and minimal clinical improvement with conventional therapy, ASCENIV was explored. On Day 10 of hospitalization, the patient received ASCENIV 500 mg/kg. Over the next several days, his clinical status improved. The patient was subsequently extubated, weaned to room air, and discharged from the hospital. Just prior to discharge, his IgG total was 210 mg/dL. In this patient, no adverse effects were observed after administration of ASCENIV. He has remained on monthly immunoglobulin replacement therapy for hypogammaglobulinemia and is followed closely by a clinical immunologist.

Patient 2 was a 15-month-old boy with history of recurrent viral and RSV infections (**Table 1**). Upon presentation, he was positive for RSV on viral RT-PCR. The patient was admitted to the PICU for respiratory failure requiring intubation. The patient received high-dose intravenous (IV) steroids, HFOV, nitric oxide, broad-spectrum antibiotics. Respiratory viral panel testing during his hospitalization demonstrated continued RSV, as well as rhinovirus and enterovirus, positivity.

Primary immune evaluation (Day 8) was suspicious for specific antibody deficiency (SAD), although the official diagnosis could not be made due to his inability to receive Pneumovax based on his age. The patient had received all age-appropriate pneumococcal vaccinations, but no pneumococcal titers were evident at initial evaluation. He also demonstrated progressive T-cell lymphopenia, thought to be partially attributable to steroid use since admission. Genetic testing revealed several variants, one including a heterozygous pathological mutation in CARD9 at p.Val13Ile. The mutation is associated with autosomal dominant and recessive immune disease and increased susceptibility to opportunistic fungal infections (2). Considering his low T-cell count and VUS in CARD9, he was started on prophylaxis for pneumocystis jirovecii pneumonia (PJP).

His presentation was concerning for an underlying immune dysregulation disorder because of the clinical context. He did not follow the standard disease course for an RSV infection, which is 5-7 days of respiratory distress followed by improvement afterwards. He was intubated for a prolonged period, which is not typical in standard severe RSV infections. In cases where the RSV course requires intubation, prolonged requirement for respiratory support, and persistent viral positivity, it raises concern for immune dysregulation (9).

The patient’s clinical status improved marginally after 500 mg/kg conventional IVIG was given on Day 18. Three days later, the patient received ASCENIV 500 mg/kg. By Day 28, the patient’s clinical condition improved. Two sequential viral tests, rapid detection and RT-PCR demonstrated clearance of RSV. No adverse effects were observed after administration of ASCENIV in this patient. He was to remain on immunoglobulin replacement therapy until he could be re-evaluated for SAD. Additionally, the patient demonstrated sustained improved T-cell counts outside of the acute care setting. PJP prophylaxis was ultimately discontinued.

Patient 3 was a 5-year-old girl with an extensive medical history including SAD with frequent viral infections and pneumonia requiring multiple PICU admissions, cerebellar ataxia, and moderate persistent asthma (**Table 1**). The patient presented to the hospital with increased work of breathing, decreased activity and poor urine output over the previous two days. Initial evaluation revealed RSV positivity using viral RT-PCR. Considering her prior history of rapid respiratory decline in the setting of respiratory infections and her current increased respiratory effort, the patient was subsequently admitted to the PICU on bilevel positive airway pressure (BiPAP).

She was started on a five-day course of azithromycin along with high-dose IV steroids. Other supportive measures including continuous albuterol, chest high frequency oscillations and a combination of an inhaled steroid and long-acting bronchodilator were also implemented.

On Day 4 of hospitalization, the immunology team was consulted due to her history of SAD. While admitted she received conventional IVIG, but despite this, the patient clinically decompensated requiring intubation and mechanical ventilation. On Day 6, viral RT-PCR testing again showed positivity to RSV; and ASCENIV 500 mg/kg was given. Three days later, repeat RSV rapid detection testing was negative. No adverse effects were observed after administration of ASCENIV in this patient. She was successfully extubated on Day 11 of hospitalization and gradually improved until discharge on Day 20. The patient has remained on IVIG therapy since discharge and has been clinically stable without any further hospitalizations.

## Discussion

RSV is the most common etiology of bronchiolitis in young children. While most children clinically improve with care at home, RSV remains the leading cause of hospitalization among infants aged 12 months or less (5). Up to forty percent of children will experience lower respiratory tract infections during the initial RSV infection. Infection with RSV does not provide long-term immunity, with reinfections common throughout a patient’s lifetime (10).

There have been prevention strategies for RSV in high-risk children, including palivizumab. Palivizumab is a monoclonal antibody that provides passive immunoprophylaxis to infants born at less than 32 weeks of gestation and/or in children less than 2 years-of- age with cardiopulmonary disease. It is given as monthly injections during the traditional months of the RSV season. The recent observation of the disruption in RSV seasonality since the COVID-19 pandemic has presented challenges with administration of palivizumab. Some have posed the consideration of giving more than the standard five consecutive doses (11). There are additional monoclonal antibodies being evaluated, but they are still in clinical trials (12). Most recently in May of 2023, Arexvy became the first RSV recombinant vaccine approved by the FDA for adults 60 years and older (13). Currently, there are no approved vaccinations for the pediatric population.

Treatment for RSV disease is mainly supportive. In patients who have an immune dysregulation and a severe course of RSV, ribavirin and standard IVIG have been utilized. Ribavirin is a nucleoside analogue and is the only FDA-approved antiviral medication for the treatment of RSV. There have been concerns, though, about its high cost and effectiveness related to administration difficulties (11, 14). Regarding IVIG, there has historically been an unmet need for more direct neutralizing antibodies within the IVIG products. Therefore, ASCENIV poses a new consideration for possibly optimizing management.

ASCENIV is an approved IVIG that is manufactured from blending normal source plasma with plasma from donors that possess high antibody titers against a range of respiratory viral pathogens of concern. The efficacy and safety of ASCENIV has been documented in a Phase 3 study conducted in 59 patients. Patients were between the ages of 2 to 75 years with primary immunodeficiency disease. Each patient received ASCENIV 300-800 mg/kg every three or four weeks for 12 months. There were zero serious bacterial infections, thus meeting the primary endpoint. It was found in the study that the titer of anti-RSV neutralizing antibody increased by 5.47-fold and by 6.79-fold in subjects who received greater than 500 mg/kg (15).

In an investigation into the presence of antibody titers of ASCENIV versus other immunoglobulin therapies, ASCENIV was compared in aggregate to 10 different lots of commercially available standard polyclonal IVIG products. ASCENIV demonstrated consistently higher titers to nine respiratory pathogens assessed, including RSV, parainfluenza 1,2,3, influenza A and B, coronavirus OC43 and 229E, and human metapneumovirus. The mean titers of all antibodies were 1.5-fold higher in ASCENIV compared with standard IVIG and ranged from 1.4 to 1.9-fold higher depending upon the particular virus (16). Although it has high RSV anti-viral titers, ASCENIV does not currently possess the FDA-approved label for the treatment and prevention of RSV in immunocompromised individuals.

A study published by Falsey et al. (14), reported a series of 15 patients with immunosuppression/dysregulation and severe RSV infection. The patients ranged from 2 months-of-age to 71 years-of-age and received the ASCENIV precursor, RI-001, after failing standard care. Standard care included a variation of corticosteroids, standard IVIG, ribavirin, and/or palivizumab. In the analysis, the pre- and post-infusion neutralizing titers were measured. Each patient had a minimum of 4-fold increase in RSV-neutralizing antibody titers 5-10 days after the infusion. Seventy three percent of patients clinically improved and were discharged from the hospital.

This case series presents similar clinical findings. All three pediatric patients discussed were found had to have, or previous had, known immune abnormalities. Each patient experienced a severe course of RSV, which resulted in intubation and mechanical intubation. The concern for underlying immune dysregulation was validated because each presentation had a prolonged course of illness and failed best supportive care. In a large retrospective analysis at a tertiary care center, 73% of those who were admitted to PICU and required intubation from RSV had an immunodeficiency, hematologic, or oncologic process (17). ASCENIV was explored in each case as a possible adjunct to the current management strategy. Each patient did subsequently recover from the RSV illness and were discharged from the hospital. Although viral load studies were not collected, RSV RT-PCR was obtained on some of the patients and did demonstrate clearance. There was a mice model study done by Boukhvalova et al. (18) that demonstrated viral load reduction after treatment with the second generation product, RI-002 (ASCENIV). The mice were made immunodeficient with repeat exposure to cyclophosphamide, and subsequently infected with RSV. When RI-002 was given, it was observed that viral replication was inhibited, and that pulmonary inflammation and epithelial hyperplasia was minimized compared to the non-treated group.

The primary aim of this case series is two-fold. First is to propose the potential benefit of ASCENIV, in conjunction with best standard care, in the treatment of patients with severe RSV infections and immune dysregulation. It is important to note that true causality of ASCENIV in these patients’ outcomes cannot be determined as that would need to be done in randomized controlled trials. Increasing awareness of this IVIG form may propel interest in future studies that investigate this specific question.

The second aim is to describe the minimal side effects of ASCENIV administration in pediatric patients. While ASCENIV has been approved for use in adults and adolescents (12-17 years of age), the safety and effectiveness of ASCENIV in children has not been well established in clinical trials (19). Compared to other IVIG forms, ASCENIV holds similar risks, including hypersensitivity reactions, aseptic meningitis, hemolysis, transmissible infectious agents, and interactions with medications. The top side effects reported include headache, sinusitis, nausea, and gastroenteritis (19). Each patient in the case series tolerated ASCENIV well and did not have any reported side effects. Falsey et al. (14) reported a similar finding with 53% of their cohort being less than 18 years-of-age.

## Conclusion

The severity of RSV bronchiolitis in the three cases described raised concern for contributing primary immune dysregulation. Each patient did demonstrate immune abnormalities and suffered from clinical deterioration despite best standard care. This propelled the medical team to pursue ASCENIV as an additional treatment option. One dose of ASCENIV (500mg/kg) was given during the hospitalization which, in conjunction with the other medical interventions and natural course of the infection, resulted in the patients ultimately making a full recovery. None of the patients suffered from the adverse side effects described on the IVIG label. The potential causality of ASCENIV in the improved clinical outcomes should be explored as conclusions cannot not be reliably drawn from a case series. Future directions may include randomized controlled trials to further investigate the question of ASCENIV being an effective RSV treatment in this less well studied age cohort.

## Data availability statement

The original contributions presented in the study are included in the article/supplementary material. Further inquiries can be directed to the corresponding author.

## Ethics statement

Written informed consent was obtained from the individual(s) for the publication of any potentially identifiable images or data included in this article.

## Author contributions

All authors listed have made a substantial, direct, and intellectual contribution to the work and approved it for publication.
